# The Impact of Goat Milk Pretreatment with Pulsed Electric Fields on Cheese Quality

**DOI:** 10.3390/foods12234193

**Published:** 2023-11-21

**Authors:** Carla Barbosa, Alberta Araújo, Paulo Fernandes, Alexandre Romão, Manuel Rui Alves

**Affiliations:** 1 CISAS, Instituto Politécnico de Viana do Castelo, Rua Escola Industrial e Comercial de Nun’Álvares, 4900-347 Viana do Castelo, Portugal; alberta@estg.ipvc.pt (A.A.); paulof@estg.ipvc.pt (P.F.); mruialves@estg.ipvc.pt (M.R.A.); 2 LAQV-REQUIMTE, Associated Laboratory for Green Chemistry—Network of Chemistry and Technology, Faculdade de Farmácia, Universidade do Porto, 4050-313 Porto, Portugal; 3 CEB—Centre of Biological Engineering, Universidade do Minho, 4710-057 Braga, Portugal; 4 Escola Superior de Tecnologia e Gestão, Instituto Politécnico de Viana do Castelo, Av. do Atlântico 644, 4900-348 Viana do Castelo, Portugal; alexandre.m.romao@gmail.com

**Keywords:** pulse electric fields, PEF, goat cheese, goat milk

## Abstract

To reduce the microbial load in goat’s milk, which is less thermally stable than cow’s milk, an alternative processing method was used in this study. This involved treating the milk with pulsed electric fields (PEFs) (at 10 kV·cm^−1^, with 50 µs pulses for 3 Hz) and then heat-treating it at 63 °C for 6.0 s, as well as using heat treatment alone at 75 °C for 3.4 s. Cheeses were made using both types of milk treatment, and samples were collected after 5, 15, and 25 days of ripening for DNA extraction and purification, followed by high-throughput sequencing on the MiSeq Illumina sequencing platform. Analysis of the bacterial populations in the two types of cheese using various diversity indices revealed no significant differences in species richness and abundance, although there was a trend for the PEF-treated cheese to have a less diverse set of species with an uneven distribution of relative abundance. However, when examining the composition of the microbial communities in the two types of cheese using Weighted UniFrac analysis and Analysis of Similarities, there were significant differences in the presence and abundance of various species, which could have implications for the development of starter cultures. Concerning physicochemical properties (pH, aw, moisture content, total acidity and L, and a and b color parameters), the results also reveal that, generally, no significant differences were found, except for the color parameter, where cheeses treated with PEF demonstrated more whiteness (L) and yellowness (b) during ripening. Sensory scores for typicity (caprylic, goaty, and acetic) increased over time, but between treatments, only small differences were perceived by panellists in cheese with 5 days of ripening. Concerning texture firmness and cohesiveness, the PEF+HT samples presented lower values than the HT samples, even over storage time. In general, concerning quality parameters, similar behavior was observed between the treatments during the ripening period.

## 1. Introduction

Cheesemaking procedures starting with raw milk are continued by rennet clotting with or without microbial starters, cutting and curd work, followed by whey drainage. Fresh cheese is then submerged in salt brine and ripened for 3 to 4 weeks. Besides standardization, the industrial production of traditional cheese seeks to ensure safety by minimizing contamination and the survival of undesirable microorganisms, namely, pathogenic ones. It must not be forgotten that complex and often unpredictable microflora dynamics are responsible for important organoleptic issues among cheeses [1].

There are several types of cheese, depending on the technologies employed as well as on the type of milk used. In fact, cheese is a product obtained from the processing of milk from many different animal origins, such as cow, sheep, and goat, and in some countries, even from buffalo and camels. Although there are cheeses that are produced with raw milk, most industrially produced cheeses are obtained from pasteurized milk for reasons of public health, assuring safety and microbiological stability.

The pasteurization of goat milk usually relates to heat treatment with the purpose of inactivating pathogenic microorganisms and consequently reducing the native flora, which is important from an economic and commercial point of view. Temperatures must be controlled to avoid important changes in the properties of the milk, particularly its color and taste, and consequently, a loss of typicity reflected in its cheese. Several authors refer to heat treatments as harmful to nutritional and sensory quality [2,3,4].

The heat treatment conditions widely implemented in the dairy and cheese industry are low-temperature long-time pasteurization at 63 °C for 30 min, low-temperature short-time pasteurization at 72 °C for 10 s, and high-temperature pasteurization at temperatures higher than 80 °C for a brief time, usually for seconds.

Mainly due to unwanted organoleptic effects of heat processing, some alternative approaches to obtain microbiologically safe milk have been attempted, such as high-pressure and pulsed electric fields (PEFs). Raw milk cheeses possess unique flavor and texture characteristics not obtainable in cheeses from pasteurized milk. PEFs as a non-thermal pasteurization technique can maintain the original characteristics of some constituents, which can be an advantage over traditional heat processing [5]. Applying mild temperatures during treatment can avoid the destruction of sensitive compounds [6]. Hence, PEFs can be an attractive technology for milk processing in cheesemaking, since, in addition to security, they also preserve several sensory attributes.

Treatment with pulsed electric fields is an emerging technology that is gaining great importance in the dairy industry as a pretreatment technique in the pasteurization process, enhancing microorganisms and enzyme inactivation at temperatures lower than those used in typical heat treatments, such as ultra-high-temperature (UHT) treatment [7]. As a consequence, greater amounts of certain heat-sensitive nutrients, such as vitamins, proteins, and other compounds, are preserved.

PEFs are a food-processing technology that uses short pulses of high-voltage electricity to selectively damage microbial and plant cell membranes [8]. This can be used to preserve and enhance the flavor of foods, including cheese. A PEF, an electric field applied at ambient temperature for a short time (in the range of microseconds), causes a potential difference across the cell membrane, inducing a sharp increase in membrane conductivity and permeability and affecting cell viability [9]. 

The main device of PEF equipment is a food treatment chamber with electrodes that are responsible for generating an electric field. The strength of this field is dependent on the distance between the electrodes (which usually consist of two parallel plates [10]), which determines the ability to keep the electric field uniform. The equipment can operate in batch or continuous mode, but a batch system is more homogeneous in its electric field distribution [11]. The levels of electric field strengths can range from 10 to 80 kV·cm ^−1^. 

Although most of the published research on the effects of PEFs on microbial viability was performed using high-power PEFs (>20 kV·cm^−1^), there have also been some applications of mild PEFs (≅10 kV·cm^−1^) in the dairy industry [12,13,14,15,16]. According to Gentès and collaborators [17], depending on the applied PEF conditions, cell membrane changes can be permanent or reversible. Thus, after milk treatment, some microorganisms can recover and induce important organoleptic characteristics. This recovery depends on the milk composition, native microflora, and storage conditions [17,18].

Although food safety is an extremely important aspect, nutritional and sensory quality are currently the factors that most influence consumers, whose concern has inspired many scientific studies and industry developments, and the use of PEFs in milk as an alternative to thermal pasteurization has been the subject of several approaches [19,20,21].

Taking into account the previous points, the main objective of the present study was to examine the impacts of PEFs combined with the mild heating treatment of milk on cheese quality compared with cheeses made from milk pasteurized by conventional heat treatment methods. Special attention was paid to important organoleptic characteristics and physical aspects (texture and color). Sensory evaluation was conducted throughout the ripening process, alongside the analysis of microbial populations in the two types of cheeses.

## 2. Materials and Methods

### 2.1. Cheese Manufacture and Experimental Design

Raw goat milk was kindly provided by a traditional cheese dairy farm, Prados de Melgaço (Melgaço, Portugal). Samples of raw goat milk were obtained within 2 h after milking, collected in low-density polyethylene (LDPE) bags and transported at 4 ± 1 °C, directly to the Food Processing and Engineering Laboratory, Viana do Castelo, Portugal. Samples were stored at 4 ± 1 °C until use, within 12 h. Raw goat milk was divided into four batches, two batches for the conventional milk heat treatment process (HT) and the other two batches for PEF + mild heat-treated milk (PEF+HT). In total, 40 L of raw goat milk were separated into 4 equal batches of 10 L, which were assigned to the two treatments, HT and PEF+HT, used in the production of two different batches of cheese (two replicate processes for each type of treatment). The resulting curd from each 10 L batch was divided into equal portions, resulting in approximately 10 × 100 g cheese samples, making a total of forty samples.

Two batches of raw goat milk were pasteurized in an Armfield FT74XTA HTST/UHT system (Armfield, Hampshire, UK) at 75 °C for 3.4 s and the other two batches were heat treated at 63 °C for 6 s after being subjected to a PEF pre-treatment (PEF+HT). Both conditions were previously established, so that a reduction equivalent to 5 log in *Listeria monocytogenes* ATCC 13932, previously spiked on raw milk, could be obtained. PEF treatments were performed using a laboratory-scale PEF unit (EPULSUS^®^-LPM1A-10 by EnergyPulse Systems, Lda, Lisbon, Portugal). The tests were carried out in continuous mode, using a fixed electric field strength of 10 kV·cm^−1^, a 50 µs pulse width, 3 Hz and a flow rate of 2.92 L·h^−1^ using a peristaltic pump (Watson Marlow 313S, Marlow, UK).

**Figure 1:**
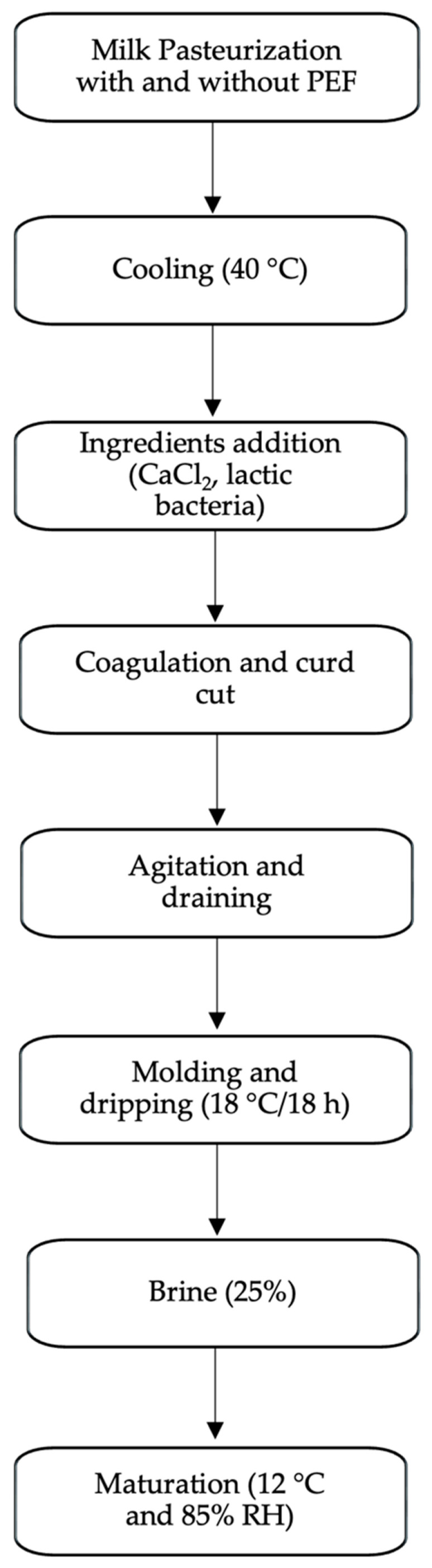
Flowchart of cheese production.

Using batches of goat milk pasteurized in different ways (HT and PEF+HT), the production of ripened cheese was carried out, simulating the industrial process summarized in the flowchart in Figure 1.

All cheeses produced were stored and ripened at 12 °C and 85% relative humidity (RH) for a period of 25 days, and were analyzed at 5, 15 and 25 days. Following this procedure, samples were assigned specific codes: A refers to milk treated with conventional heat treatment (HT), while B denotes milk pretreated with pulsed electric fields followed by heat treatment (PEF+HT). These letters are then followed by the sampling time (5, 15 or 25), and subsequent numbers 1 or 2 have been used to differentiate between batch replicates. For instance, “cheese B.15.2” refers to the cheese produced with PEF+HT-treated milk, aged for 15 days, belonging to batch replicate 2. Codes A.0.1, A.0.2, B.0.1 and B.0.2 have been employed for the microbiological analysis of curd samples. Throughout all tables and figures, samples will be identified by these designated codes.

### 2.2. Physicochemical Analysis

Final goat cheeses aged 5, 15 and 25 days were analyzed for pH (AOAC 981.12-2016), moisture content (AOAC 948.12-2002) and titratable acidity (AOAC 947.05-2016). Water activity determinations were performed using a LabSwift-aw water activity meter (Novasina, Lachen, Switzerland). All the analyses were carried out in triplicate, taking into account duplicate samples for each treatment (with and without PEF pretreatment).

### 2.3. Microbiological Analysis of Milk and Cheese

A 25 g portion of cheese, approximately 1 cm deep, was removed from several locations within a block of cheese, without contacting the cheese rind. These samples were weighed aseptically into sterile stomacher bags and diluted 10 times with Buffered Peptone Water, BPW (Liofilchem srl, Roseto degli Abruzzi, Italy). Sample homogenization was performed in a stomacher (Laboratory Blender Stomacher 400; Seward, London, UK) for 1 min. Serial decimal dilutions were made in maximum recovery diluent (Liofilchem srl, Roseto degli Abruzzi, Italy) and used for the inoculation of culture media. Serial decimal dilutions of milk were also prepared in the maximum recovery diluent. The quantification and detection of microorganisms was performed according to standardized methods, namely, ISO 4833-1:2013 [22] for the enumeration of microorganisms at 30 °C in Plate Count Agar (Scharlau, Barcelona, Spain), ISO 21528-2:2017 [23] for the enumeration of Enterobacteriaceae in VRBG (Scharlau, Barcelona, Spain), and ISO 16649-2:2001 [24] for the enumeration of *Escherichia coli* in Tryptone Bile X-glucuronide, TBX (Biomerieux, Craponne, France). The detection of *Salmonella* spp. and *L. monocytogenes* was performed after the sample’s initial suspension in BPW for 24 h at 37 °C and performed according to ISO 6579-1:2017 [25] and ISO 11290-1:2017 [26], respectively.

### 2.4. DNA Extraction and Processing

DNA extraction and purification from milk and cheese was performed according to Rocha et al. [27]. Three replicate tubes containing 6 mL each of milk were pelleted at 13,000 rpm (Hettich, Tuttlingen, Germany) for 10 min. The supernatant was discarded, and the pellet was resuspended in DNA/RNA Shield^TM^ solution (ZymoResearch, Irvine, CA, USA). For cheese, three replicate 400 mg portions were aseptically taken from the interior of each cheese and transferred to a tube containing a DNA/RNA Shield^TM^ solution. All replicate milk and cheese samples in DNA/RNA Shield^TM^ solution were homogenized in a bead beater (Benchmark Scientific, Sayreville, NJ, USA) for 6 min at maximum speed and stored at −20 °C until further processing. Total DNA was extracted from each sample replicate using ZymoBIOMICS^TM^ DNA Miniprep Kit (ZymoResearch, Irvine, CA, USA), following the manufacturer’s instructions. The eluted DNA was cleaned and concentrated using a DNA Clean and Concentrator^TM^ Kit (ZymoResearch, Irvine, CA, USA), following the manufacturer’s instructions. Finally, purified DNA from three independent extractions of each sample was pooled together and utilized for subsequent steps. The quantification of extracted and purified DNA was performed by fluorimetry using Qubit 3.0 (Thermo Fisher Scientific, Waltham, MA, USA). PCR amplification with the extracted, purified, and pooled DNA was performed using primers targeting the V3 to V4 hypervariable regions of the 16S rRNA gene for bacterial identification using the primers (connected with barcodes) 341F (5′-CCTAYGGGRBGCASCAG-3′) and 806R (5′- GGACTACNNGGGTATCTAAT-3′). The PCR products of proper size were selected by 2% agarose gel electrophoresis, normalized, pooled, end-repaired, A-tailed and further ligated with Illumina adapters. Libraries were sequenced on a paired-end Illumina platform to generate paired-end raw reads at Novogene Company Limited (Cambridge, UK).

### 2.5. Bioinformatics and Sequencing Results Analysis

Paired-end reads were assigned to samples based on their unique barcodes and truncated by cutting off the barcode and primer sequences. Paired-end reads were merged using FLASH (V1.2.7) [28]. Quality filterings on the raw tags were performed to obtain high-quality clean tags [29] according to the Qiime (V1.7.0) quality-controlled process [30].

The tags were compared with the reference database SILVA138 using the UCHIME algorithm [31] to detect and remove chimera sequences [32].

Sequences analysis was performed using Uparse software (Uparse v7.0.1090) [33], using all the effective tags. A threshold of ≥97% similarity was defined to assign sequences to the same OTUs. For each representative sequence, Qiime (Version 1.7.0), using the Mothur method, was performed against the SSUrRNA of the SILVA138 Database with species annotation at each taxonomic rank [34,35]. To obtain the phylogenetic relationship of all OTUs’ representative sequences, MUSCLE (Version 3.8.31) was used [36]. The abundance information for OTUs was normalized using a standard of sequence number corresponding to the sample with the lowest number of sequences.

### 2.6. Nucleotide Sequences Accession Number

Raw reads were deposited in the SRA database under BioProject PRJNA928317.

### 2.7. Sensory Evaluation

The sensory evaluation was carried out by an internal panel consisting of 10 assessors evaluating the goat cheese. Samples’ sensory profiles were constructed according to Stone et al. (2020) [37] implementing a Quantitative Descriptive Analysis technique (QDA), focusing mainly on taste and odor attributes. The list of attributes was developed by the panel members by consensus, based on a lexicon for goat cheese [4].

All assessors underwent comprehensive training in cheese profiling, with a particular emphasis on cheeses made from goat milk. This training encompassed a wide variety of cheese types, with a special reference to cheeses made from goat milk. Several goat cheeses, and some cow and blended cheeses, were used to teach assessors about goat cheese characteristics. Panel training sessions were held to familiarize assessors with the language and products under investigation. Following this initial stage, 7-point intensity scales were defined for each attribute, and verbal anchors were also defined for the lowest (grade 1) and highest (grade 7) intensity. Reference standards were defined or specially produced as verbal anchors for training assessors. The final attribute scoring list was agreed (Table 1) and assessors were asked to carry out a taste session to validate it.

As shown in Table 1, sensory attributes were grouped by external and mass appearance, flavor, taste, and residual perceptions.

Taste sessions took place at 5, 15 and 25 days after the goat cheese was produced. Three-digit coded samples were presented in a random order within and between assessors at room temperature. Water and apples were provided to assessors to rinse the palate between samples. Sensory analysis was conducted in duplicate trials, and there were also duplicates given by each assessor.

### 2.8. Instrumental Texture Evaluation

The texture assessment of goat cheese was carried out using a Texture Analyzer TAXT2i (Stable Micro System, Godalming, Surrey, UK) equipped with a 5 kg load cell. The Texture Exponent Software version 6.1.20 was used to record the data while performing a TPA (Texture Profile Analysis). Samples were cut into cubes (15 mm edge) and were subjected to a two-bite compression test using a P-75 plate compression probe. The temperature of cheese samples was stabilized at room temperature (20 ± 1 °C) before testing. Test conditions were set as: pre-test speed (2.0 mm·s^−1^), test speed (1 mm∙s^−1^), post-test speed (2.0 mm·s^−1^) with a resting period of 3 s between cycles, and a data acquisition rate of 200 points/s. The following textural parameters were obtained from the TPA results (according to Bourne, M.C [38]): *hardness*, expressed in N, is the maximum force detected in the first compression cycle; *adhesiveness*, expressed in N, is the maximum negative force recorded between the two compression cycles, which is proportional to the corresponding negative area; *cohesiveness* is calculated as the ratio between the area of the 2nd compression cycle to the area of the first compression cycle (Area2/Area1); *springiness* is measured as the ratio of the distance traveled by the probe until it finds the food surface in the second compression cycle, to the same distance measured in the first cycle; *gumminess*, expressed in N, is given by hardness × cohesiveness; *chewiness* is determined as hardness × cohesiveness × springiness, and expressed in N; *resilience* is calculated as the ratio of upward energy in the first cycle to the downward energy in the same cycle.

### 2.9. Instrumental Color Evaluation

Color evaluation over ripening time was performed using the CIE (“Commission Internationale de l’éclairage”) color spaces in the format of L*, a* and b*. The L* value indicates lightness with values ranging from 0 to 100, a* represents the green (−) to red (+) component, and b* indicates the blue (−) to yellow (+) component. Results have been expressed as an average of ten to twenty measurements performed on both inside and outside surfaces [39,40].

### 2.10. Data Analysis

General data analyses were carried out using the R version 4.2.3 (R foundation for statistical computing). Data mining was carried out with principal component analysis (PCA) to investigate differences between treatments, and to correlate the main characteristics and their changes along the ripening time. PCA was performed using the Autobiplot.PCA function built into R language by M. Rui Alves [41]. The analysis of diversity was based on the normalized output data in order to calculate the alpha diversity metrics such as Shannon [42], observed features, Faith phylogenetic diversity [43], and Evenness, and to determine metrics for beta diversity including Jaccard distance [44], Bray–Curtis dissimilarity [45], and unweighted and weighted UniFrac [46,47] and create principal coordinates analysis plots (PCoA).

In order to identify differentially abundant taxa between different samples with possible biological significance, a Linear Discriminant Analysis (LDA) of Effect Size (LEfSe) was executed on a Galaxy computational tool (http://huttenhower.sph.harvard.edu/galaxy/, accessed on 19 December 2022).

## 3. Results

Forty cheeses were manufactured, with half of them produced using milk heated to 75 °C for 3.4 seconds, and the other half using milk treated with PEF and then heat-treated at 63 °C for 6 seconds. Both methods resulted in a 5-log_10_ reduction in *L. monocytogenes*. The cheeses were ripened for 25 days, and were analyzed on days 5, 15, and 25. Additionally, the microbiome of the respective cheese curds was analyzed shortly after their formation.

### 3.1. Cheese Microbiological Analysis

All cheese samples produced in the laboratory were controlled during the ripening stage in terms of the enumeration of total microorganisms at 30 °C, *E. coli* and *Enterobacteriaceae,* as well as detecting the presence of *Salmonella* spp. and *L. monocytogenes*. The results are summarized in Table 2.

In relation to *E. coli* and *Enterobacteriaceae*, no growth was observed in any of the cheeses tested. Therefore, the results presented in Table 1 are expressed in accordance with the detection limit of the method used. Furthermore, neither *L. monocytogenes* nor *Salmonella* spp. were detected after a 24 h enrichment period. As is evident from Table 1, there is no significant difference in the total count of mesophilic microorganisms present in the cheese samples, regardless of whether they were produced with conventionally heat-treated (HT) milk or milk subjected to PEF pretreatment followed by heat treatment (PEF+HT).

### 3.2. Analysis of the Sequencing Results

Curd (samples A.0.1, A.0.2, B.0.1 and B.0.2) and cheese bacterial microbiomes for all repining times were analyzed by 16S amplicon high-throughput sequencing. In general, the rarefaction curves showed a stabilizing tendency, indicating the sufficient sampling of microbial communities, and a high Goods Coverage was obtained, reflecting the quality of the sequencing and the relatively accurate representation of the bacterial community in the cheese samples (Table S1).

#### 3.2.1. Diversity Metrics

The type of milk treatment used to produce the cheeses did not significantly affect the α-diversity metrics for bacterial microbiota (Table 3). However, a trend toward statistical significance in the Sympson index was observed (*p* = 0.05908), suggesting that cheeses made with PEF+HT-treated milk exhibited a higher abundance of a few dominant species and a lower abundance or absence of other species compared to cheeses produced exclusively with HT milk. No other significant differences were observed between HT and PEF+HT cheese samples.

Although a similar overall diversity was found between the two groups (HT and PEF+HT), the analysis of β-diversity, using Weighted and Unweighted UniFrac, showed that the two groups of samples differ significantly in terms of microbial community compositions, with distinct relative abundances of various microbial taxa (Table 4).

**Figure 2:**
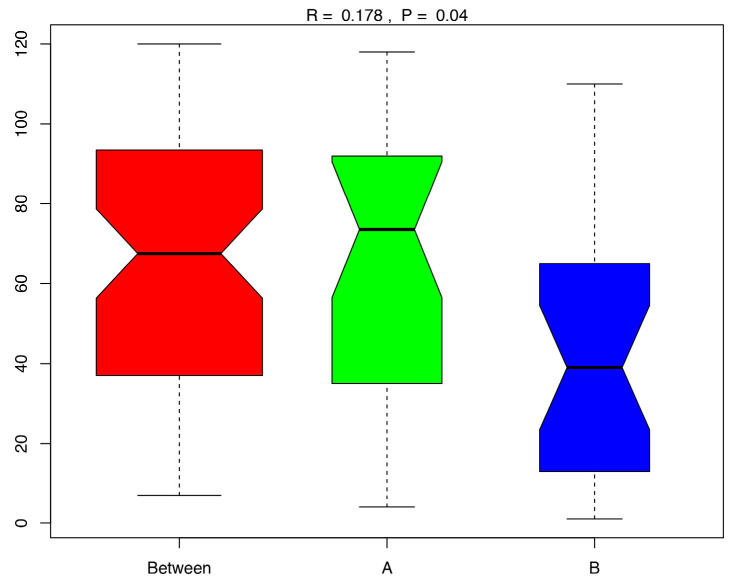
ANOSIM diagram. A—HT cheeses; B—PEF+HT cheeses.

An analysis using principal coordinates analysis (PCoA) was conducted to compare the microbiomes of HT and PEF+HT cheese samples (Figure S1), and the Analysis of Similarities (ANOSIM) showed that, in terms of bacterial flora composition, cheeses of type HT and type PEF+HT are significantly different (*R* = 0.178, *p* = 0.04), as can be seen in Figure 2.

#### 3.2.2. Bacterial Composition of Cheeses of Type HT and PEF+HT

**Figure 3:**
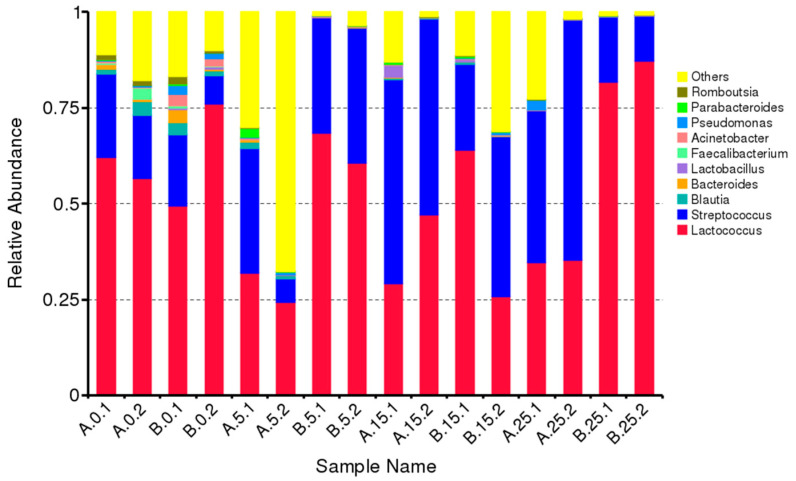
Barplot of relative abundances (% of dominant sequences) of the ten most abundant genera. “Others” represents a total relative abundance of the rest genera besides the top 10.

The top ten genera, based on the relative abundance of species in each sample (considering both HT and PEF+HT cheeses), were used to create the barplot presented in Figure 3. *Streptococcacea* is, by far, the most abundant family (81.4% of all OTUs), composed essentially of two genera, *Lactococcus* (52.2%) and *Streptococcus* (29.2%). The rest of the bacterial flora are present in much smaller amounts, in relative terms.

**Figure 4:**
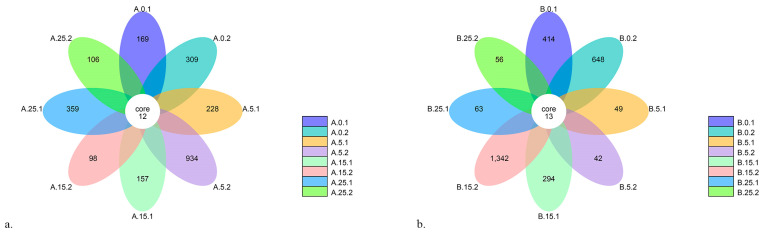
Flower diagrams: (**a**) HT, (**b**) PEF+HT. Each petal in the flower represents a sample. The core number in the center stands for the number of OTUs that are common to all samples, while the number in the petals represents the unique OTUs only present in the respective sample.

A comparison using flower diagrams (Figure 4) revealed both similarities and differences in the bacterial species present in cheeses of type HT and PEF+HT. The core microbiome of both HT and PEF+HT cheeses is basically composed of the same genera of bacteria, namely, *Streptococcus*, *Lactococcus*, *Mannheimia*, *Romboutsia*, *Pseudomonas* and *Acinetobacter*. However, the presence of the genus *Escherichia* (specifically *E. coli)* is only common to all cheeses produced with HT milk.

**Figure 5:**
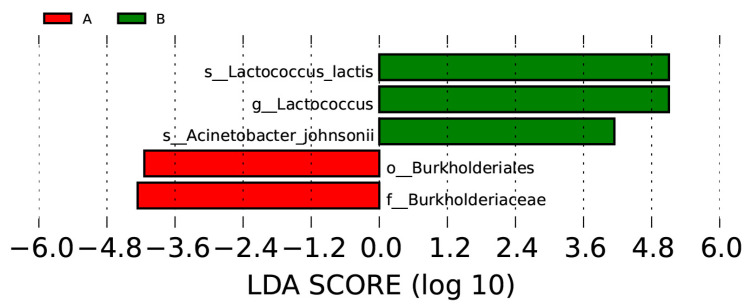
Linear Discriminant Analysis (LDA) scores computed for bacterial features differentially abundant in cheeses of type A (HT) and type B (PEF+HT).

Some divergent taxa between the two groups of cheeses appear to be statistically significant, as indicated by an LEfSe analysis on the data (Figure 5). The most relevant features are related to *Acinetobacter johnsonii* and *Burkholderiaceae*, with *Lactococcus lactis* being particularly prominent. In relative terms, *Lactococcus lactis* is more abundant in cheeses produced with PEF+HT milk compared to cheeses produced with HT milk, as demonstrated by the LDA scores (*p* = 0.014).

### 3.3. Cheese Physicochemical Analysis

The physicochemical evaluation of goat cheeses produced with milk pasteurization (HT) and with PEF and mild heating (PEF+HT), over the ripening period of 25 days, is summarized in Table 5.

**Figure 6:**
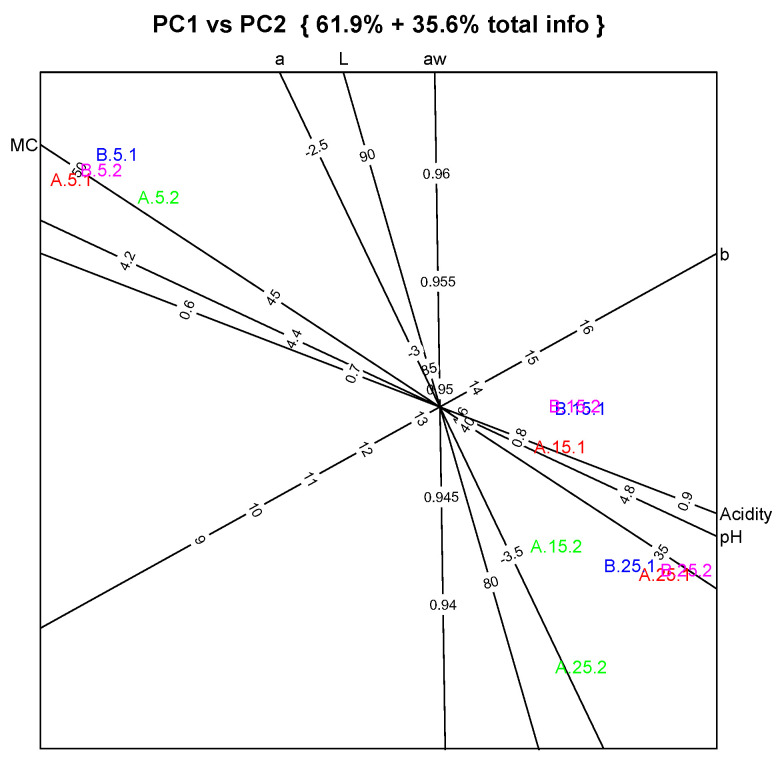
Autobiplot applied to the plane of principal components 1 vs. 2 derived from physicochemical data used for the evaluation of cheeses produced with HT (samples A) and PEF+HT (samples B) goat milk. Output shows the axes representing important parameters equipped with measuring scales, enabling us to interpret results directly in terms of measured values. Sample coding: 5, 15 and 25 refer to ripening days; 1 and 2 refer to replicated processes. MC = moisture content %; acidity is expressed as lactic acid %; L, a and b are color parameters (L*, a* and b*, respectively).

A quick inspection of Table 5 can lead to the conclusion that the main physicochemical parameters do not show relevant differences between samples or throughout ripening. To visualize the data presented in Table 5 in a more convenient way, a principal component analysis (PCA) was performed. Furthermore, for the PCA to be as informative and accurate as possible, the analysis was performed with the function “Autobiplot.PCA”, which applies a biplot to the PCA displays. The biplot applied to the plane of principal component 1 and 2 is shown in Figure 6.

The biplot represents almost 98% of the total information contained in Table 5. The biplot is interpreted as follows: an imaginary straight line is projected orthogonally from any sample point to a variable axis. The point where the projection line and the variable axis meet is the sample value with respect to that variable. For example, sample A.5.2 projects orthogonally to pH ≈ 4.2, acidity ≈ 0.55, moisture content ≈ 47%, a* ≈ −2.5, a_w_ ≈ 9.57, L* ≈ 90 and b* ≈ 11. Therefore, this type of PCA allows the interpretation of results in terms of original variables and sample values obtained in practical work, avoiding inconvenient interpretations in terms of latent variables and relative values. An important feature of this type of biplot is that a biplot axis is drawn on the graph only if its mean standard predictive error (MSPE) is small, which means that the readings taken relative to the axis in question are very accurate.

It is also observed that variables pH and acidity are positively correlated, and both negatively correlate with moisture content, which is verified by the proximity of the respective axes in the biplot. The variables a*, L* and, to a lesser extent, a_w_ are all positively correlated, while the parameter b* appears to be uncorrelated with the other variables (the b* axis lies more or less orthogonal to the other axes).

Following the reasoning presented in the previous example with cheese A.5.2, the biplot clearly shows that samples are clustered by ripening time, with no evident differences between treatments. Samples with 5 days of ripening (A.5.x and B.5.x) are clustered on the lefthand upper side of the graph, and project towards the higher values of the moisture content axis (around 50%), lower acidity (≈0.5) and pH (≈4.1), and higher a_w_ values (≈0.96). They also have high values of L*. On the other hand, samples with 25 days of ripening (A.25.x and B.25.x) are located on the opposite corner of the plot, presenting higher values along the axes corresponding to pH (≈4.85) and to titratable acidity (≈0.9), and lower values along the a_w_ axis (0.940 ≤ a_w_ ≤ 0.945), and they present low values of L*. The color parameters a* and b* assume very small values in all samples, indicating a white color typical of goat cheese, which loses brightness throughout ripening. Samples with 15 days of ripening (A.15.x and B.15.x) are projected in the center and to the right of the graph, in a more or less intermediate position, although closer to the ripened samples. The main difference between samples with 15 and 25 ripening days is seen in a_w_, with a significant decrease from 0.95 to below 0.94, and with slightly higher a* and lower L*. As expected, the cheese samples with 5 days of maturation, still considered fresh cheese, presented much higher moisture content values than the remaining samples. Concerning milk treatment influence, PCA does not show any relevant discriminating pattern.

The changes referred to in the previous paragraph are associated with the expected loss of water during ripening and the fermentative activity of lactic acid microbiota. It is important to realize that, as shown in the biplot, although some changes are small, such as those observed with parameters a* and b* (the human eye cannot perceive any color change of such a small magnitude), they are significative between ripening times, but not between milk pretreatments.

### 3.4. Cheese Texture and Sensory Evaluation

Sensory evaluation is essential when validating products and procedures within the scope of quality control, and as a method of evaluating whether changes introduced in a product are perceived by consumers, as well as possible impacts in the acceptability of products by consumers [39,48,49].

**Figure 7:**
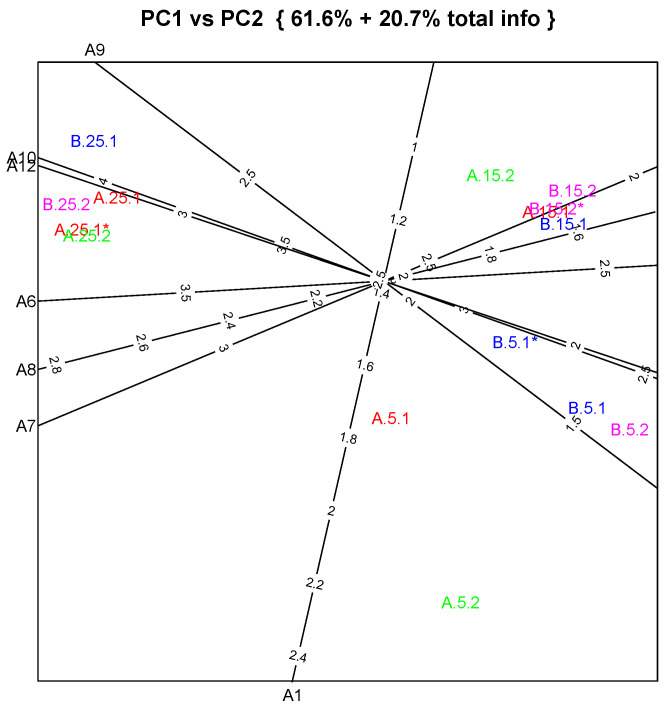
Principal component analysis applied to sensory characteristics data of cheeses produced with HT (A) and PEF-HT (B) goat milk. Output shows the important variables (extracted from eigen vectors) equipped with corresponding attribute scales. Sample coding: 5, 15 and 25 refer to ripening days, and 1 and 2 refer to replicate and processes; variables A1, A6, A7, A8, A9, A10 and A12 are attributes, as in Table 1.

The sensory assessors considered goat cheeses produced with milk pretreated with PEF to be very similar to those made with HT milk only. The data analysis outlines the expected significant differences among ripening times, showing that the judges noticed the evolution of some cheese attributes over storage time. Between treatments, these differences are smaller, as was expected since the heat treatment was mild in both cases, showing that PEF had no significative influence. The PCA of sensory data was performed with the Autobiplot.PCA function for R software, obtaining the biplot presented in Figure 7.

PCA generally shows that assessors do not differentiate cheese by pasteurization procedure, but that they make a sharp distinction depending on the ripening time. Figure 7 shows samples with different ripening times clustering together, although projected on the plot separately by ripening time. Both types of cheese produced with 25 days of ripening differ considerably from the other samples, as regards sour taste (A12 ≈ 3.5 points) and more intense animal flavor (A10 ≈ 4 points), as well as the goaty flavor notes, such as vinegar (A7 > 3.5 points), floral (A8 > 2.8 points), and sour milk (A9 ≈ 2.75 points). The last two are attributes typical of goat cheese conferred by caprylic, caproic and butyric acids, which are the predominant short-chain fatty acids in goat milk.

Sensory evaluation also revealed that small differences were perceived, essentially concerning external color (A1) together with sour milk flavor (A9), mainly concerning cheeses with 5 days of storage. At this stage, the samples still undergo large changes typical of an early maturation stage, reflected in a great within-group dispersion, as seen in the biplot, which means that although physicochemical parameters are similar, as discussed previously, some organoleptic differences are well perceived by sensory assessors. After this initial maturation time, the samples become more homogenous: with 15 days ripening, the samples are projected together in the upper right corner of this PCA biplot, and on the opposite side of the biplot are the samples with 25 days ripening. According to the assessors’ results, this clear separation is mainly due to flavor attributes, namely, lactic (A6), vinegar (A7) and floral (A8) flavor aspects.

Considering texture assessment, cheese samples were subjected to a two-cycle compression test, known as TPA (texture profile analysis), and texture parameters were calculated.

The results shown in Table 6 reveal that, in general terms, firmness, chewiness and gumminess increase over time, while elasticity and adhesiveness decrease, as expected and as is in line with the moisture content trend already discussed. Changes in these textural parameters can be explained by water loss during the ripening stage. Several types of cheese undergo similar changes to those reported by Benedito et al. [50].

The results also show that samples produced with PEF+HT milk generally presented lower firmness values than HT milk. The same trend was observed for the resilience parameter, although this was not as evident. This observation was also reported by Gentès and co-workers, in their review on the effects of the PEF treatment of milk on subsequent cheesemaking properties [17]. Several studies reported slight differences between PEF-treated milk and raw milk, even though these studies were performed with higher (almost two times higher) PEF voltage. As concerns cohesiveness, which is a very important parameter in cheese texture, the results show that the type of treatment was not relevant to the differentiation of samples.

**Figure 8:**
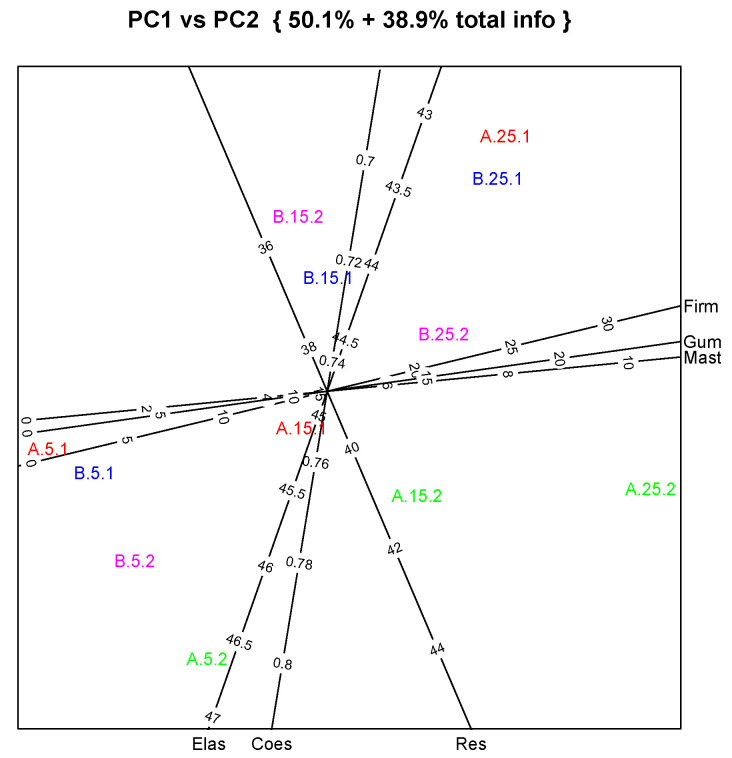
Principal component analysis applied to data of texture characteristics of cheeses produced with HT (sample A) and PEF-HT (sample B) goat milk. Output shows the important variables (extracted from eigen vectors) equipped with corresponding original variable scales. Sample coding: 1 and 2 refer to replicates and 5, 15 and 25 refer to ripening days. Variables’ abbreviations: Firm, Ads, Elas, Coes, Mast, Gum and Res for firmness, elasticity, cohesiveness, chewiness, gumminess and resilience, respectively.

As for previous analyses, a PCA biplot applied to texture data is presented in Figure 8. As observed by the assessors during the sensory analysis, the samples are quite similar, except when considering differences in ripening time. Cheese samples with 25 days of ripening time are projected towards the right side of the plot, with higher values of firmness, gumminess, and chewiness. On the opposite side are those with just five days of ripening. It can also be observed that these samples, with less maturity, present variations between them. These differences are due to the fact that the gels are still forming, and are doing so at different rates even between different batches of the same production procedure. The texture analysis also revealed that replicates of cheese HT samples (coded as A.5.2; A.15.2 and A.25.2) behave differently from the other batch of cheese produced with the same type of milk treatment (HT) due to some problems that occurred during the forming step, especially in the draining step before brine immersion. In this PCA output, it can be observed that these samples are projected separately at the bottom of the plot.

## 4. Discussion

Pulsed electric fields have been shown to be effective in microbial inactivation. However, the use of PEF for controlling microbial flora, particularly bacterial, usually requires the use of PEF with high-intensity electric fields [51]. Many factors such as the type of microorganism, size and shape of the cells, and growth conditions can influence the efficiency of PEF in microbial inactivation [51]. The smaller the cell size, the smaller the membrane potential induced by the action of the electric field, and a higher microbial resistance to PEF treatments is reached [51,52]. The small size of bacteria is a challenging factor when using PEF in bacterial inactivation, particularly when using low- or moderate-intensity PEF. However, using PEF in combination with other treatments, such as thermal methods, has been proven effective. In fact, low-intensity PEF (<10 kV·cm^−1^) may not be sufficient to cause the desired reduction in the microbial flora, but it affects a large number of cells, making them more sensitive to subsequent treatments, such as thermal treatment. This is what was attempted in this study. In fact, it was possible to ensure a 5-log reduction in the initial microbial load of the milk (indicated by the reduction in *L. monocytogenes* artificially spiked in a milk sample) using a PEF treatment followed by a heat treatment at a moderately high temperature and for a very short time (63 °C for 6 s). Despite the appropriate reduction in microbial load, the sensitivity of different microorganisms to PEF is different [52], making it necessary to evaluate the use of this methodology for obtaining milk for cheese production. Cheese is a complex food product whose physicochemical and organoleptic characteristics depend greatly on the evolution dynamics of a group of microorganisms that will be responsible, over time, for a series of biochemical phenomena with implications in terms of proteolysis, lipolysis, and the production of secondary metabolites, which will have a significant impact on the final quality.

The microbiological analysis of the produced cheeses showed acceptable results in terms of microbiological quality, with no detection of *Salmonella* spp. or *L. monocytogenes*. The levels of mesophilic microorganism were very similar in cheeses produced with thermally treated milk or milk treated with PEF followed by heat treatment. However, it is important to have a broader understanding of the potential influence of milk treatment on the global microbial population throughout the cheese ripening process (after 5, 15, and 25 days, corresponding roughly to the time used for the commercialization of these types of cheeses with different ripening times) and the potential implications for their physicochemical and organoleptic characteristics.

The analysis of the microbiome by 16S amplicon high-throughput sequencing did not reveal significant differences between cheeses produced with thermally treated milk and milk pretreated with PEF. The main α-diversity indices, namely, Simpson, ACE, Chao1, and Shannon, of HT and PEF+HT cheeses are not proven to be significantly different, indicating identical species richness and abundance, although with a tendency for cheese produced with milk treated with PEF to show a lower diversity of species, and with a more uneven distribution of relative abundance. However, the composition of the microbial communities of the two types of cheese, namely, the presence/absence of each species and their relative abundances, are effectively different, as revealed by a Weighted UniFrac analysis (*p* = 0.02612) and by an Analysis of Similarities (ANOSIM, *p* = 0.04). Cheeses were produced using a commercial starter culture consisting mainly of *Lactococcus*, *Streptococcus*, and *Lactobacillus*. It is not surprising, therefore, that some of these genera are among the most abundant in all cheeses. As revealed by a Linear Discriminant Analysis, the relative amounts of *Lactococcus* are significantly different between the two types of cheese, being significantly higher in cheeses produced with milk treated with PEF followed by a heat treatment. As the addition of the starter culture was performed after the milk treatment, only phenomena of competition between different bacterial species can justify the greater growth of this species in cheeses produced with milk treated with PEF. As already mentioned, these types of cheeses actually have a lower bacterial diversity. *L. lactis* is one of the main species with functional relevance in cheese production and is widely used in starter cultures [53]. Due to its ability to produce significant amounts of lactic acid, contribute to milk acidification, and produce enzymes during ripening, it plays a crucial role in the production of compounds that significantly impact the organoleptic and textural characteristics of cheese [54]. However, despite the significant differences in the relative abundance of *L. lactis* between cheeses produced with heat-treated milk or PEF-treated milk, this does not seem to have an influence on the organoleptic characteristics of the cheeses produced.

The presence of *Acinetobacter johnsonii*, although in very low relative abundances, is more evident in cheeses produced with PEF+HT milk, and particularly in curds, probably indicating a greater resistance of this species to pulsed electric fields. While cheeses made from various types of milk exhibit variations in their bacterial community structures, they share a very similar core microbiome. This core microbiome mainly comprises *Streptococcus*, *Lactococcus*, *Mannheimia*, *Romboutsia*, *Pseudomonas*, and *Acinetobacter* species. *Streptococcus* and *Lactococcus* are the dominant genera, whereas *Pseudomonas*, *Romboutsia*, and *Acinetobacter* are present in considerably lower proportions on average.

It is also worth noting the presence of *E. coli*, although with very low read counts in all cheeses produced with thermally treated milk, unlike cheeses treated with PEF, where the presence of *E. coli* was not recorded. No colonies were detected on the TBX medium, but this is not necessarily incompatible with this result, since it must be considered that an enumeration method with a detection limit of 10 CFU/g was used. 

Regarding the organoleptic properties and according to Briggs (2003) [55], when applied to cheese production, PEF alone can improve the flavor profile by facilitating the release of volatile compounds responsible for the aroma and taste in ripened cheeses. However, according to Gentés [17], if combined with mild HT, this will result in higher levels of residual antimicrobial proteins, which, in addition to assuring safety, will cause a ripening profile similar to that of HT as regards flavor. Assessors easily distinguished samples with different ripening times mostly because of the intensification of typical goat cheese odors. The assessors also found PEF-treated goat’s milk cheese to be slightly less intense in bitterness and sourness, which is in line with the findings of other authors reporting that PEF can also be used to reduce the bitterness and astringency of some cheeses [17,56].

In relation to the physical characteristics of cheese, mainly in terms of the results obtained for texture, it appears that they were similar to those reported by Sepulveda-Ahumada et al. (2000) [9] on the effect of PEF on the texture attributes of cheddar cheese: hardness, springiness, adhesiveness, cohesiveness and flavor. As observed in this work, the use of PEF in milk used for goat cheese production increases hardness, springiness, and cohesiveness, in comparison with cheese made from raw milk. This is in line with other authors, who stated that PEF can improve the texture and consistency of cheese by modifying the protein structure. This can lead to a smoother, creamier texture in soft cheeses, and a firmer texture in hard cheeses [57].

According to Gentés and collaborators [17], the ideal situation would be to apply a mild treatment to the milk, capable of destroying pathogens without affecting organoleptic characteristics [58,59]. This implies a minimal effect on compounds responsible for generating the special “raw milk” flavors in ripened cheeses. This justifies the growing importance of PEF in cheesemaking, which, in addition to ensuring safety, as with heat treatment, will not affect the organoleptic properties of the cheese to the same extent.

## 5. Conclusions

The results indicate that PEF can be used as a complementary method in addition to mild thermal pasteurization as a processing technique ensuring food safety, and maintaining the physicochemical and organoleptic attributes of goat cheese, since no significant differences were observed between the cheeses, although some textural aspects such as hardness and cohesiveness were slightly different in PEF-treated samples. In this study, PEF+HT samples showed similar behaviors when compared to HT samples during ripening in terms of organoleptic quality and microbial safety.

Overall, PEF is a promising technology for use in enhancing the flavor and texture of cheese, while also extending its shelf life.

Although there are limited studies on the effects of PEF on milk compounds and their use in cheesemaking, those that do exist justify the interest in using PEF in cheesemaking. Thus, more research is needed to understand the effects of PEF on different cheese types in order to benefit the microbiome and optimize processing parameters.

## Figures and Tables

**Table 1:** Sensory attributes used to perform QDA and corresponding codes used in data analysis.

Tasting phase	Group of Attributes	Specific Attributes	Codes
Visual	External	Color	A1
	appearance	Slits	A2
	Mass appearance	Color	A3
		Eyes	A4
		Rind thickness	A5
Olfato-gustative	Flavor	Lactic	A6
		Vinegar (acetic ac.)	A7
		Floral (caprylic ac.)	A8
		Sour milk (butyric ac.)	A9
		Animal stable	A10
		Typicity/goaty	A11
	Taste	Sour	A12
		Bitter	A13
	Other	Residual bitterness	A14
		Residual goaty/capric	A15

**Table 2:** Microbiological analysis of cheese.

Sample	Treatment *	Ripening Time (days)	Enumeration of Microorganisms at 30 °C (log_10_ (CFU·g^−1^))	Enumeration of *Enterobacteriaceae* (log_10_ (CFU·g^−1^))	Enumeration of *E. coli* (log_10_ (CFU·g^−1^))	Detection of *L. monocytogenes* (in 25 g)	Detection of *Salmonella* spp. (in 25 g)
A.5	HT	5	6.71 ± 0.0275	<1	<1	Not detected	Not detected
B.5	PEF	5	6.65 ± 0.0437	<1	<1	Not detected	Not detected
A.15	HT	15	6.65 ± 0.0966	<1	<1	Not detected	Not detected
B.15	PEF	15	6.68 ± 0.0509	<1	<1	Not detected	Not detected
A.25	HT	25	6.64 ± 0.370	<1	<1	Not detected	Not detected
B.25	PEF	25	6.82 ± 0.0186	<1	<1	Not detected	Not detected

* Treatment of goat milk: HT—heat treatment at 75 °C for 3.4 s; PEF+HT—PEF at 10 k·Vcm^−1^, 50 μs pulses and 3 Hz frequency + heat treatment at 63 °C for 6 s.

**Table 3:** Summary of α-diversity significance data (HT vs. PEF+HT cheese).

Simpson		ACE		Chao 1		Shannon		Observed Species	
*t*-Test *p*-Value	Two-Wilcoxon	*t*-Test *p*-Value	Two-Wilcoxon	*t*-Test *p*-Value	Two-Wilcoxon	*t*-Test *p*-Value	Two-Wilcoxon	*t*-Test *p*-Value	Two-Wilcoxon
0.05908	0.06496	0.3694	0.2786	0.2989	0.3282	0.1696	0.1304	0.3376	0.3282

**Table 4:** Summary of β-diversity significance data: HT vs. PEF+HT cheese.

Weighted-Unifrac		Unweighted-Unifrac	
*p*-Value	Two-Wilcoxon	*p*-Value	Two-Wilcoxon
0.02612	0.15268	0.18588	0.34885

**Table 5:** Some physicochemical characteristics of cheese batches produced with HT and PEF+HT milk, over ripening time (5, 15 and 25 days). MC = moisture content %; AL = acidity expressed as lactic acid %; L*, a* and b* are the CIELAB color parameters corresponding to lightness, green–red and blue–yellow, respectively.

Treatment *	Time (days)	CODES	pH	a_w_	MC (%)	AL (%)	L*	a*	b*
HT	5	A.5.1	4.12 ± 0.01	0.961	50.41 ± 1.04	0.53 ± 0.01	91.53 ± 1.03	−2.32 ± 0.22	9.91 ± 0.85
HT	5	A.5.2	4.19 ± 0.01	0.957	46.59 ± 0.6	0.53 ± 0	89.85 ± 0.82	−2.44 ± 0.21	11.05 ± 0.51
PEF+HT	5	B.5.1	4.08 ± 0.02	0.962	50.92 ± 0.4	0.53 ± 0	91.43 ± 0.98	−2.4 ± 0.25	11.12 ± 1.26
PEF+HT	5	B.5.2	4.17 ± 0.03	0.962	49.18 ± 0.85	0.53 ± 0	92.01 ± 0.96	−2.29 ± 0.23	10.36 ± 0.87
HT	15	A.15.1	4.72 ± 0.02	0.947	38.85 ± 0.66	0.83 ± 0.02	80.31 ± 3.21	−3.34 ± 0.23	14.61 ± 1.6
HT	15	A.15.2	4.78 ± 0.04	0.945	38.8 ± 0.33	0.85 ± 0.01	79.95 ± 1.14	−3.33 ± 0.17	14.15 ± 0.48
PEF+HT	15	B.15.1	4.73 ± 0.02	0.947	38.27 ± 0.61	0.84 ± 0.01	83.97 ± 1.07	−3.4 ± 0.13	15.11 ± 0.53
PEF+HT	15	B.15.2	4.76 ± 0.01	0.946	37.2 ± 0.95	0.84 ± 0	83.53 ± 1.93	−3.17 ± 0.24	14.9 ± 0.95
HT	25	A.25.1	4.84 ± 0.01	0.938	34.83 ± 0.3	0.89 ± 0.01	80.71 ± 1.54	−3.87 ± 0.27	15.35 ± 1.13
HT	25	A.25.2	4.86 ± 0.02	0.941	34.39 ± 1.5	0.89 ± 0.01	77.2 ± 3.11	−3.88 ± 0.25	12.97 ± 0.79
PEF+HT	25	B.25.1	4.8 ± 0.02	0.938	33.75 ± 1.73	0.88 ± 0	81.18 ± 1.96	−3.63 ± 0.21	14.79 ± 0.99
PEF+HT	25	B.25.2	4.86 ± 0.01	0.938	35.09 ± 0.39	0.89 ± 0.01	77.56 ± 5.21	−3.57 ± 0.31	15.89 ± 2.07

**Table 6:** Texture characteristics of cheese samples produced with HT and PEF+HT milk at different stages of ripening (5, 15 and 25 days). Values reported as mean ± standard deviation.

Treatment	Time (Days)	CODES	Firmness (N)	Adhesiveness (N)	Elasticity	Cohesiveness	Chewiness	Gumminess	Resilience
HT	5	A.5.1	4.62 ± 0.97	−0.27 ± 0.08	46.21 ± 0.77	0.78 ± 0.02	1.65 ± 0.31	3.57 ± 0.7	37.45 ± 1.64
HT	5	A.5.2	6.19 ± 1.38	−0.16 ± 0.08	46.23 ± 0.4	0.8 ± 0.02	2.29 ± 0.51	4.95 ± 1.11	43.34 ± 1.62
PEF+HT	5	B.5.1	4.22 ± 0.85	−0.22 ± 0.07	45.83 ± 0.44	0.77 ± 0.02	1.49 ± 0.28	3.25 ± 0.62	38.71 ± 1.75
PEF+HT	5	B.5.2	3.07 ± 0.66	−0.13 ± 0.05	46.73 ± 2.01	0.78 ± 0.02	1.11 ± 0.23	2.39 ± 0.52	39.67 ± 2.92
HT	15	A.15.1	11.41 ± 2.2	−0.1 ± 0.07	44.9 ± 0.67	0.75 ± 0.03	3.85 ± 0.7	8.57 ± 1.58	39.11 ± 1.82
HT	15	A.15.2	17.08 ± 2.45	−0.09 ± 0.07	44.91 ± 0.7	0.76 ± 0.02	5.8 ± 0.77	12.93 ± 1.74	41.18 ± 1.71
PEF+HT	15	B.15.1	13.81 ± 3.28	−0.12 ± 0.07	43.78 ± 0.99	0.72 ± 0.04	4.35 ± 1.04	9.93 ± 2.36	37.01 ± 2.35
PEF+HT	15	B.15.2	14.3 ± 4.12	−0.14 ± 0.08	43.48 ± 0.97	0.72 ± 0.03	4.4 ± 1.02	10.38 ± 2.54	36.15 ± 1.96
HT	25	A.25.1	31.05 ± 6.54	−0.18 ± 0.13	43.27 ± 0.79	0.69 ± 0.02	9.31 ± 2.09	21.5 ± 4.75	36.25 ± 2.32
HT	25	A.25.2	32.6 ± 8.95	−0.1 ± 0.08	45.06 ± 0.64	0.76 ± 0.02	11.17 ± 2.97	24.8 ± 6.67	42.52 ± 2.51
PEF+HT	25	B.25.1	25.11 ± 4.37	−0.07 ± 0.07	43.37 ± 1.79	0.7 ± 0.05	7.59 ± 1.47	17.47 ± 3.15	36.06 ± 3.29
PEF+HT	25	B.25.2	20.86 ± 4.79	−01 ± 0.08	44.27 ± 0.55	0.73 ± 0.02	6.74 ± 1.64	15.22 ± 3.63	38.61 ± 1.6

## Data Availability

Raw Illumina sequencing reads are available in the SRA database under BioProject PRJNA928317. Specific data concerning physicochemical, sensory, and textural aspects can be shared upon request.

## Supplementary Materials

The following supporting information can be downloaded at: https://www.mdpi.com/article/10.3390/foods12234193/s1, Figure S1: Principal Coordinate Analysis based on Weighted Unifrac distance, Table S1. Summary of α-diversity metrics.
