# Supplementary material for: The Impact of Goat Milk Pretreatment with Pulsed Electric Fields on Cheese Quality
Source: Foods. 2023 Nov 21;12(23):4193. doi: 10.3390/foods12234193 (PMC10706457; doi:10.3390/foods12234193)
Supplement: Supplementary file 1 [file foods-12-04193-s001.zip › foods-2663956-supplementary.pdf]

Table S1. Summary of α-diversity metrics

Sample	Observed species	Shannon	Simpson	Chao1	ACE	Goods_Coverage
A.0.1	378	2.396	0.568	461.081	482.121	0.998
A.0.2	534	2.997	0.652	918.169	1050.337	0.994
B.0.1	779	3.621	0.719	922.922	1044.174	0.995
B.0.2	893	2.143	0.417	1413.627	1630.045	0.990
A.5.1	1183	4.469	0.792	1280.927	1313.556	0.995
A.5.2	2559	7.944	0.936	2931.185	2913.480	0.989
B.5.1	144	1.067	0.441	196.059	209.751	0.999
B.5.2	224	1.418	0.509	257.735	267.839	0.999
A.15.1	473	2.667	0.633	564.969	557.002	0.998
A.15.2	246	1.237	0.518	477.000	445.550	0.997
B.15.1	595	2.309	0.543	681.443	741.962	0.997
B.15.2	1588	4.177	0.757	1766.125	1913.315	0.991
A.25.1	1765	3.828	0.723	2288.772	2383.691	0.987
A.25.2	197	1.206	0.483	244.458	267.397	0.999
B.25.1	173	0.844	0.303	205.766	218.134	0.999
B.25.2	122	0.655	0.227	183.091	238.480	0.999

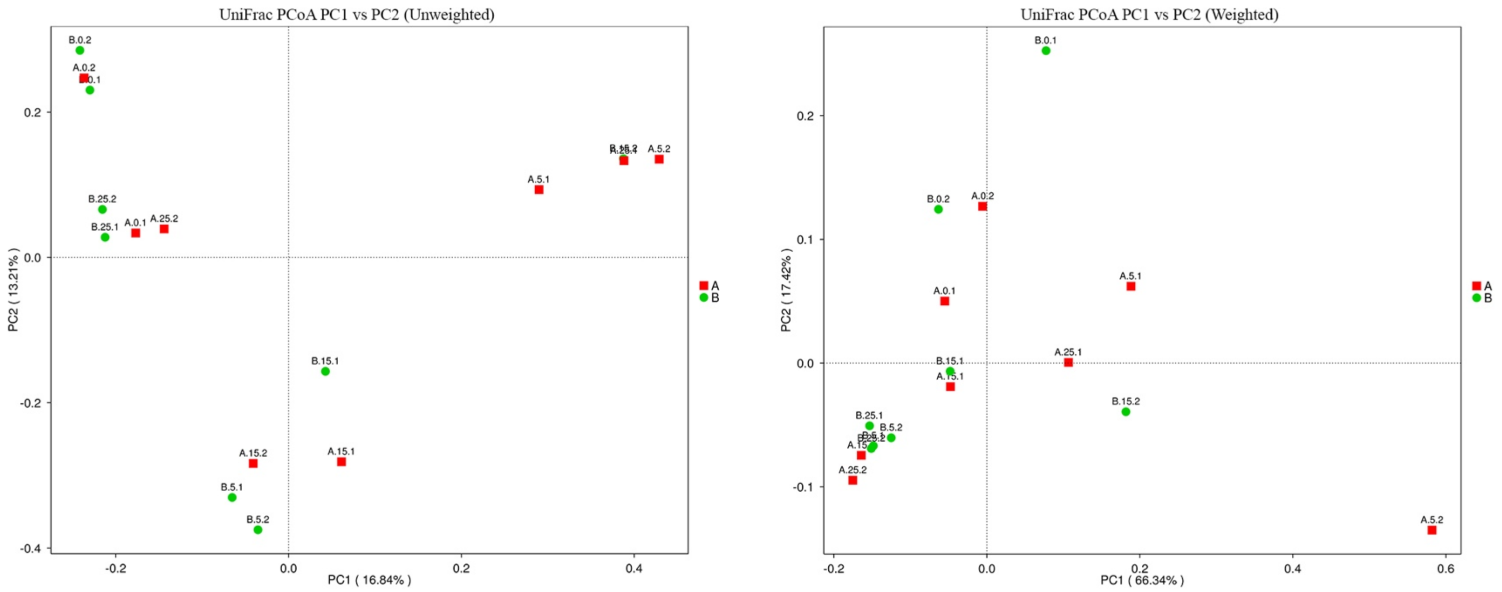

Figure S1: Principal Coordinate Analysis based on Weighted Unifrac distance
